# Supplementary material for: Early tissue damage and microstructural reorganization predict disease severity in experimental epilepsy
Source: eLife. 2017 Jul 26;6:e25742. doi: 10.7554/eLife.25742 (PMC5529108; doi:10.7554/eLife.25742)
Supplement: Figure 2—source data 1. — Quantitative values of T2 measurements are listed for individual mice (saline-injected: N12, NP13, NP17, NP28, NP29; kainate-injected: NP10, NP11, NP14, NP25, NP26, NP27, NP31, NP34) and longitudinal time points (pre, 1d, 4d, 8d, 16d, 31d following injection). DOI: http://dx.doi.org/10.7554/eLife.25742.007 [file elife-25742-fig2-data1.docx]

| **Parameter** | **saline-injected mice** | | | |  |  | **kainate-injected mice** | | |  |  |  |  |  |
| --- | --- | --- | --- | --- | --- | --- | --- | --- | --- | --- | --- | --- | --- | --- |
| **T_2_-weighted imaging** | | |  |  |  |  |  |  |  |  |  |  |  |  |
|  |  | |  |  |  |  |  |  |  |  |  |  |  |  |
| T_2_ (CA1) | NP12 | | NP13 | NP17 | NP28 | NP29 | NP10 | NP11 | NP14 | NP25 | NP26 | NP27 | NP31 | NP34 |
| pre | | 10.97618 | 10.44801 | 10.72581 | 10.88061 | 10.9156 | 10.10729 | 11.01611 | 10.11051 | 10.52657 | 11.11818 | 11.29122 | 10.25653 | 9.974009 |
| 1d | 10.66517 | | 9.958719 | 10.38156 | 10.7722 | 10.87898 | 10.89364 | 12.46934 | 13.10165 | 14.99202 | 13.97047 | 13.12624 | 13.07348 | 13.40129 |
| 4d | 10.06062 | | 10.00634 | 10.26437 | 10.69415 | 10.72057 | 10.22293 | 10.19135 | 9.915945 | 12.73643 | 11.51137 | 10.27758 | 10.91094 | 11.06666 |
| 8d | 10.49581 | | 10.37648 | 10.31272 | 10.53495 | 11.27678 | 10.04635 | 10.69916 | 9.86718 | 10.61256 | 10.65379 | 10.28598 | 10.97198 | 10.58975 |
| 16d | 11.24927 | | 9.883635 | 10.61159 | 10.62624 | 10.7731 | 10.92337 | 10.85993 | 10.16613 | 10.09903 | 9.805709 | 9.650801 | 10.26484 | 10.00146 |
| 31d | 10.62068 | | 10.34511 | 9.860616 | 10.83016 | 10.48191 | 10.40232 | 10.59326 | 9.767027 | 11.27201 | 9.667801 | 10.00895 | 9.907178 | 9.758342 |
|  |  | |  |  |  |  |  |  |  |  |  |  |  |  |
| T_2_ (DG) | NP12 | | NP13 | NP17 | NP28 | NP29 | NP10 | NP11 | NP14 | NP25 | NP26 | NP27 | NP31 | NP34 |
| pre | 10.99095 | | 10.70062 | 10.81957 | 11.16243 | 11.20032 | 10.24224 | 11.07445 | 10.11272 | 11.01533 | 11.06483 | 11.19057 | 10.61148 | 10.25369 |
| 1d | 10.67151 | | 10.27139 | 10.63175 | 10.6666 | 11.21119 | 10.81207 | 11.72948 | 11.21031 | 12.55745 | 11.87493 | 11.6309 | 11.51766 | 12.43011 |
| 4d | 10.38879 | | 10.34428 | 10.50678 | 11.02606 | 11.17166 | 10.19288 | 10.52499 | 10.22966 | 11.20394 | 11.43653 | 11.10806 | 11.17405 | 10.91984 |
| 8d | 10.73136 | | 10.81324 | 10.5457 | 10.57212 | 11.62212 | 10.5657 | 11.67803 | 11.42038 | 12.12825 | 11.25394 | 11.3961 | 12.01772 | 11.67881 |
| 16d | 11.35558 | | 10.3245 | 11.27451 | 10.83486 | 11.19581 | 11.58419 | 11.99804 | 12.24869 | 12.44054 | 11.42268 | 10.98474 | 11.97533 | 11.50816 |
| 31d | 11.0129 | | 10.63446 | 10.34421 | 11.01544 | 11.31205 | 10.83114 | 11.37763 | 11.68199 | 13.3631 | 11.11526 | 11.46874 | 11.46794 | 11.55768 |
|  |  | |  |  |  |  |  |  |  |  |  |  |  |  |

**Figure 2 - source data 1: Summary of T_2_ metrics.** Quantitative values of T_2_ measurements are listed for individual mice (saline-injected: N12, NP13, NP17, NP28, NP29; kainate-injected: NP10, NP11, NP14, NP25, NP26, NP27, NP31, NP34) and longitudinal time points (pre, 1d, 4d, 8d, 16d, 31d following injection).
