# Supplementary material for: Early tissue damage and microstructural reorganization predict disease severity in experimental epilepsy
Source: eLife. 2017 Jul 26;6:e25742. doi: 10.7554/eLife.25742 (PMC5529108; doi:10.7554/eLife.25742)
Supplement: Figure 3—source data 1. — Quantitative values of 1H-MR spectroscopy are listed for individual mice (saline-injected: N12, NP13, NP17, NP28, NP29; kainate-injected: NP10, NP11, NP14, NP25, NP26, NP27, NP31, NP34) and longitudinal time points (pre, 1d, 4d, 8d, 16d, 31d following injection). DOI: http://dx.doi.org/10.7554/eLife.25742.010 [file elife-25742-fig3-data1.docx]

| **Parameter** | **saline-injected mice** | | |  |  | **kainate-injected mice** | | |  |  |  |  |  |
| --- | --- | --- | --- | --- | --- | --- | --- | --- | --- | --- | --- | --- | --- |
| **1H-MR spectroscopy** | |  |  |  |  |  |  |  |  |  |  |  |  |
|  |  |  |  |  |  |  |  |  |  |  |  |  |  |
| NAA | NP12 | NP13 | NP17 | NP28 | NP 29 | NP10 | NP11 | NP14 | NP25 | NP26 | NP27 | NP31 | NP34 |
| pre | 80.430 | 80.220 | 86.830 | 80.230 | 86.910 | 81.650 | 82.510 | 79.420 | 83.180 | 76.050 | 81.270 | 76.860 | 65.720 |
| 1d | 80.150 | 78.370 | 82.930 | 70.360 | 84.400 | 71.130 | 56.140 | 40.960 | 36.200 | 39.780 | 48.970 | 40.780 | 35.240 |
| 4d | 79.400 | 76.390 | 79.020 | 70.170 | 78.970 | 60.790 | 50.730 | 46.060 | 48.470 | 41.090 | 44.540 | 45.230 | 32.640 |
| 8d | 78.060 | 80.060 | 81.670 | 69.960 | 84.570 | 66.900 | 59.370 | 48.850 | 55.200 | 41.610 | 57.230 | 52.310 | 31.710 |
| 16d | 88.340 | 72.110 | 92.250 | 73.660 | 82.400 | 83.210 | 66.040 | 56.640 | 51.300 | 43.930 | 57.910 | 52.230 | 34.990 |
| 31d | 76.370 | 80.020 | 86.720 | 80.100 | 80.480 | 76.190 | 66.500 | 53.280 | 54.880 | 53.760 | 55.850 | 49.720 | 42.860 |
|  |  |  |  |  |  |  |  |  |  |  |  |  |  |
| Glu | NP12 | NP13 | NP17 | NP28 | NP 29 | NP10 | NP11 | NP14 | NP25 | NP26 | NP27 | NP31 | NP34 |
| pre | 104.290 | 104.030 | 100.960 | 99.300 | 105.820 | 104.900 | 105.960 | 99.020 | 102.800 | 93.810 | 97.580 | 100.250 | 97.720 |
| 1d | 103.130 | 97.820 | 105.140 | 98.560 | 110.690 | 97.180 | 79.170 | 65.430 | 56.630 | 61.300 | 75.570 | 64.770 | 60.740 |
| 4d | 97.610 | 103.500 | 78.420 | 94.810 | 99.430 | 85.150 | 77.540 | 76.040 | 84.510 | 55.590 | 71.310 | 77.780 | 60.990 |
| 8d | 102.640 | 105.060 | 93.750 | 91.450 | 101.990 | 90.050 | 94.020 | 71.330 | 76.310 | 44.440 | 80.920 | 76.830 | 55.360 |
| 16d | 112.750 | 101.230 | 109.760 | 93.700 | 103.480 | 105.540 | 91.860 | 70.540 | 71.150 | 56.310 | 88.520 | 77.520 | 57.040 |
| 31d | 100.760 | 105.830 | 107.410 | 87.800 | 97.920 | 91.180 | 89.610 | 69.250 | 67.950 | 61.150 | 71.800 | 70.960 | 55.970 |
|  |  |  |  |  |  |  |  |  |  |  |  |  |  |
| GABA | NP12 | NP13 | NP17 | NP28 | NP 29 | NP10 | NP11 | NP14 | NP25 | NP26 | NP27 | NP31 | NP34 |
| pre | 37.800 | 39.900 | 49.970 | 32.030 | 41.130 | 46.830 | 44.190 | 42.450 | 38.800 | 42.230 | 40.320 | 39.900 | 37.640 |
| 1d | 44.710 | 46.350 | 44.910 | 35.800 | 45.290 | 47.800 | 38.840 | 28.030 | 18.340 | 24.470 | 27.200 | 30.550 | 19.900 |
| 4d | 43.910 | 36.740 | 42.100 | 32.960 | 40.100 | 39.840 | 35.000 | 30.490 | 20.420 | 20.660 | 31.810 | 31.620 | 28.020 |
| 8d | 46.660 | 52.060 | 48.420 | 35.320 | 38.450 | 45.420 | 34.890 | 42.740 | 32.920 | 27.670 | 31.890 | 34.240 | 26.720 |
| 16d | 51.430 | 38.770 | 55.480 | 34.380 | 43.890 | 44.940 | 31.940 | 53.470 | 50.360 | 39.270 | 37.450 | 41.110 | 33.780 |
| 31d | 30.890 | 43.450 | 43.660 | 39.830 | 42.170 | 47.530 | 44.070 | 50.190 | 51.640 | 49.020 | 34.150 | 51.350 | 50.930 |
|  |  |  |  |  |  |  |  |  |  |  |  |  |  |
| Lac | NP12 | NP13 | NP17 | NP28 | NP 29 | NP10 | NP11 | NP14 | NP25 | NP26 | NP27 | NP31 | NP34 |
| pre | 19.690 | 18.580 | 14.430 | 27.110 | 11.360 | 22.160 | 18.980 | 23.720 | 27.320 | 14.510 | 14.820 | 26.440 | 12.920 |
| 1d | 21.230 | 19.510 | 23.490 | 27.920 | 22.830 | 36.840 | 53.040 | 70.780 | 179.330 | 111.750 | 59.440 | 69.670 | 0.0000 |
| 4d | 12.720 | 18.640 | 17.520 | 23.480 | 23.770 | 13.510 | 55.140 | 61.050 | 53.060 | 162.890 | 46.910 | 59.140 | 49.360 |
| 8d | 14.440 | 0.9090 | 19.080 | 12.600 | 25.640 | 0.7730 | 38.290 | 72.150 | 34.270 | 48.810 | 46.030 | 52.340 | 24.060 |
| 16d | 13.040 | 0.7400 | 22.340 | 19.320 | 16.310 | 19.030 | 21.880 | 48.090 | 76.300 | 54.370 | 23.190 | 14.690 | 44.080 |
| 31d | 13.310 | 14.370 | 24.230 | 19.570 | 18.550 | 19.270 | 23.960 | 10.580 | 52.020 | 69.470 | 19.780 | 22.900 | 0.0000 |
|  |  |  |  |  |  |  |  |  |  |  |  |  |  |
| Myoi | NP12 | NP13 | NP17 | NP28 | NP 29 | NP10 | NP11 | NP14 | NP25 | NP26 | NP27 | NP31 | NP34 |
| pre | 74.030 | 65.230 | 74.620 | 43.300 | 70.940 | 68.870 | 71.510 | 72.380 | 60.890 | 57.150 | 68.530 | 68.210 | 78.890 |
| 1d | 75.920 | 68.440 | 71.680 | 40.030 | 65.870 | 65.320 | 56.340 | 44.790 | 43.670 | 32.210 | 51.190 | 53.730 | 65.210 |
| 4d | 69.670 | 71.830 | 68.120 | 35.730 | 64.190 | 86.020 | 85.360 | 93.780 | 85.130 | 42.430 | 79.940 | 85.170 | 82.760 |
| 8d | 70.590 | 65.970 | 67.610 | 38.630 | 62.200 | 76.360 | 87.770 | 97.570 | 106.240 | 59.170 | 90.160 | 89.550 | 100.570 |
| 16d | 72.250 | 71.160 | 61.030 | 35.760 | 67.080 | 85.530 | 88.640 | 87.020 | 74.740 | 54.320 | 82.780 | 87.830 | 105.370 |
| 31d | 71.290 | 68.240 | 68.230 | 49.610 | 67.990 | 76.260 | 73.360 | 73.520 | 92.040 | 53.160 | 67.310 | 68.380 | 82.460 |

**Figure 3 - source data 1: Summary of ^1^H-MR metrics.** Quantitative values of 1H-MR spectroscopy are listed for individual mice (saline-injected: N12, NP13, NP17, NP28, NP29; kainate-injected: NP10, NP11, NP14, NP25, NP26, NP27, NP31, NP34) and longitudinal time points (pre, 1d, 4d, 8d, 16d, 31d following injection).
