# Supplementary material for: Early tissue damage and microstructural reorganization predict disease severity in experimental epilepsy
Source: eLife. 2017 Jul 26;6:e25742. doi: 10.7554/eLife.25742 (PMC5529108; doi:10.7554/eLife.25742)
Supplement: Figure 5—source data 1. — Quantitative values of DWI measurements are listed for individual mice (saline-injected: N12, NP13, NP17, NP28, NP29; kainate-injected: NP10, NP11, NP14, NP25, NP26, NP27, NP31, NP34) and longitudinal time points (pre, 1d, 4d, 8d, 16d, 31d following injection). DOI: http://dx.doi.org/10.7554/eLife.25742.013 [file elife-25742-fig5-data1.docx]

| **Parameter** | **saline-injected mice** | | |  |  | **kainate-injected mice** | | |  |  |  |  |  |
| --- | --- | --- | --- | --- | --- | --- | --- | --- | --- | --- | --- | --- | --- |
| **Diffusion-weighted imaging** | | |  |  |  |  |  |  |  |  |  |  |  |
|  |  |  |  |  |  |  |  |  |  |  |  |  |  |
| MD | NP12 | NP13 | NP17 | NP28 | NP29 | NP10 | NP11 | NP14 | NP25 | NP26 | NP27 | NP31 | NP34 |
| pre | 0.2066 | 0.2142 | 0.2104 | 0.2285 | 0.2065 | 0.2200 | 0.2124 | 0.2077 | 0.2057 | 0.2069 | 0.2069 | 0.2128 | 0.2102 |
| 1d | 0.2155 | 0.1967 | 0.1951 | 0.1985 | 0.1956 | 0.2166 | 0.2208 | 0.2128 | 0.2302 | 0.1981 | 0.2204 | 0.1993 | 0.1996 |
| 4d | 0.2072 | 0.1975 | 0.2252 | 0.2125 | 0.2166 | 0.2198 | 0.2023 | 0.2168 | 0.2230 | 0.2245 | 0.2275 | 0.2265 | 0.2227 |
| 8d | 0.2183 | 0.2065 | 0.1960 | 0.2308 | 0.2202 | 0.2442 | 0.2443 | 0.2398 | 0.2508 | 0.2418 | 0.2259 | 0.2310 | 0.2435 |
| 16d | 0.2249 | 0.2185 | 0.1985 | 0.2196 | 0.2035 | 0.2400 | 0.2427 | 0.2392 | 0.2740 | 0.2511 | 0.2395 | 0.2527 | 0.2522 |
| 31d | 0.2136 | 0.2285 | 0.2311 | 0.2163 | 0.2050 | 0.2353 | 0.2523 | 0.2376 | 0.2500 | 0.2604 | 0.2385 | 0.2414 | 0.2558 |
|  |  |  |  |  |  |  |  |  |  |  |  |  |  |
| FA | NP12 | NP13 | NP17 | NP28 | NP29 | NP10 | NP11 | NP14 | NP25 | NP26 | NP27 | NP31 | NP34 |
| pre | 0.2022 | 0.2174 | 0.2230 | 0.2354 | 0.2225 | 0.2075 | 0.2190 | 0.2000 | 0.2391 | 0.2136 | 0.2136 | 0.2084 | 0.2023 |
| 1d | 0.2085 | 0.2332 | 0.2376 | 0.2194 | 0.2423 | 0.2051 | 0.2190 | 0.2252 | 0.1960 | 0.2217 | 0.1999 | 0.2219 | 0.1937 |
| 4d | 0.2180 | 0.2573 | 0.2367 | 0.2105 | 0.2507 | 0.2083 | 0.2338 | 0.2262 | 0.2463 | 0.2351 | 0.2170 | 0.2281 | 0.2054 |
| 8d | 0.2000 | 0.2703 | 0.3131 | 0.2260 | 0.2040 | 0.2177 | 0.2218 | 0.2385 | 0.2813 | 0.2569 | 0.2274 | 0.2364 | 0.2494 |
| 16d | 0.2135 | 0.2156 | 0.2660 | 0.2098 | 0.2302 | 0.2192 | 0.2228 | 0.3065 | 0.3017 | 0.2825 | 0.2274 | 0.2644 | 0.2893 |
| 31d | 0.2030 | 0.1932 | 0.1854 | 0.2103 | 0.2401 | 0.2140 | 0.2453 | 0.2759 | 0.2505 | 0.2691 | 0.2342 | 0.2722 | 0.3143 |
|  |  |  |  |  |  |  |  |  |  |  |  |  |  |
| AD | NP12 | NP13 | NP17 | NP28 | NP29 | NP10 | NP11 | NP14 | NP25 | NP26 | NP27 | NP31 | NP34 |
| pre | 0.000758 | 0.000794 | 0.000783 | 0.000856 | 0.000773 | 0.000811 | 0.000788 | 0.00076 | 0.000778 | 0.000765 | 0.000765 | 0.000784 | 0.000769 |
| 1d | 0.000795 | 0.000739 | 0.000736 | 0.000739 | 0.000745 | 0.000792 | 0.00082 | 0.000788 | 0.000836 | 0.000737 | 0.000805 | 0.00074 | 0.000721 |
| 4d | 0.000772 | 0.000757 | 0.000848 | 0.000785 | 0.000827 | 0.000806 | 0.000761 | 0.00081 | 0.000846 | 0.000846 | 0.000843 | 0.00085 | 0.00082 |
| 8d | 0.000797 | 0.000801 | 0.000786 | 0.000858 | 0.000809 | 0.000902 | 0.000909 | 0.00091 | 0.000989 | 0.000932 | 0.000848 | 0.000876 | 0.000929 |
| 16d | 0.000831 | 0.000811 | 0.000768 | 0.000811 | 0.000769 | 0.000891 | 0.000907 | 0.000962 | 0.001097 | 0.000983 | 0.000897 | 0.00098 | 0.001002 |
| 31d | 0.000783 | 0.00083 | 0.000832 | 0.000799 | 0.000775 | 0.000867 | 0.000959 | 0.000929 | 0.000957 | 0.001003 | 0.000897 | 0.000944 | 0.001038 |
|  |  |  |  |  |  |  |  |  |  |  |  |  |  |
| RD | NP12 | NP13 | NP17 | NP28 | NP29 | NP10 | NP11 | NP14 | NP25 | NP26 | NP27 | NP31 | NP34 |
| pre | 0.000551 | 0.000567 | 0.000555 | 0.0006 | 0.000543 | 0.000584 | 0.000562 | 0.000555 | 0.000537 | 0.000549 | 0.000549 | 0.000565 | 0.000561 |
| 1d | 0.000572 | 0.000516 | 0.00051 | 0.000524 | 0.000508 | 0.000579 | 0.000584 | 0.000563 | 0.000618 | 0.000523 | 0.000589 | 0.000527 | 0.000537 |
| 4d | 0.000546 | 0.00051 | 0.000589 | 0.000564 | 0.000562 | 0.000586 | 0.00053 | 0.00057 | 0.00058 | 0.000587 | 0.000602 | 0.000594 | 0.000592 |
| 8d | 0.000584 | 0.000528 | 0.000489 | 0.00061 | 0.000586 | 0.000648 | 0.000645 | 0.000624 | 0.000634 | 0.000622 | 0.000592 | 0.000602 | 0.000632 |
| 16d | 0.000596 | 0.000578 | 0.000509 | 0.000583 | 0.000531 | 0.000635 | 0.000638 | 0.000595 | 0.000685 | 0.000638 | 0.00063 | 0.000647 | 0.000634 |
| 31d | 0.000569 | 0.000613 | 0.000624 | 0.000574 | 0.000535 | 0.000625 | 0.000655 | 0.000605 | 0.000647 | 0.00067 | 0.000625 | 0.000614 | 0.000632 |
|  |  |  |  |  |  |  |  |  |  |  |  |  |  |

**Figure 5 - source data 1: Summary of DWI metrics.** Quantitative values of DWI measurements are listed for individual mice (saline-injected: N12, NP13, NP17, NP28, NP29; kainate-injected: NP10, NP11, NP14, NP25, NP26, NP27, NP31, NP34) and longitudinal time points (pre, 1d, 4d, 8d, 16d, 31d following injection).
