# Supplementary material for: Early tissue damage and microstructural reorganization predict disease severity in experimental epilepsy
Source: eLife. 2017 Jul 26;6:e25742. doi: 10.7554/eLife.25742 (PMC5529108; doi:10.7554/eLife.25742)
Supplement: Figure 6—source data 1. — Quantitative values of dorsoventral diffusivity (dvD) measurements are listed for individual mice (saline-injected: N12, NP13, NP17, NP28, NP29; kainate-injected: NP10, NP11, NP14, NP25, NP26, NP27, NP31, NP34) and longitudinal time points (pre, 1d, 4d, 8d, 16d, 31d following injection). DOI: http://dx.doi.org/10.7554/eLife.25742.017 [file elife-25742-fig6-data1.docx]

| **Parameter** | **saline-injected mice** | | |  |  | **kainate-injected mice** | | |  |  |  |  |  |
| --- | --- | --- | --- | --- | --- | --- | --- | --- | --- | --- | --- | --- | --- |
| **Diffusion-weighted imaging** | | |  |  |  |  |  |  |  |  |  |  |  |
|  |  |  |  |  |  |  |  |  |  |  |  |  |  |
| dvD | NP12 | NP13 | NP17 | NP28 | NP29 | NP10 | NP11 | NP14 | NP25 | NP26 | NP27 | NP31 | NP34 |
| pre | 0.000669 | 0.000693 | 0.000633 | 0.000769 | 0.000615 | 0.000694 | 0.000678 | 0.000626 | 0.00062 | 0.000628 | 0.000628 | 0.000663 | 0.000665 |
| 1d | 0.00066 | 0.000577 | 0.000572 | 0.000623 | 0.000616 | 0.000701 | 0.000719 | 0.000679 | 0.000728 | 0.000624 | 0.000725 | 0.000588 | 0.000623 |
| 4d | 0.000628 | 0.000572 | 0.000698 | 0.000683 | 0.000628 | 0.000651 | 0.000632 | 0.000712 | 0.000759 | 0.000716 | 0.000696 | 0.000713 | 0.000725 |
| 8d | 0.000683 | 0.000578 | 0.000559 | 0.000734 | 0.000677 | 0.000752 | 0.000797 | 0.000805 | 0.000895 | 0.000804 | 0.000741 | 0.000774 | 0.000839 |
| 16d | 0.000688 | 0.000656 | 0.000567 | 0.000718 | 0.000655 | 0.000769 | 0.000766 | 0.000817 | 0.001017 | 0.000867 | 0.000761 | 0.000866 | 0.00089 |
| 31d | 0.000674 | 0.000716 | 0.0007 | 0.000721 | 0.000604 | 0.000752 | 0.000819 | 0.000814 | 0.00084 | 0.000862 | 0.000789 | 0.000836 | 0.000955 |
|  |  |  |  |  |  |  |  |  |  |  |  |  |  |
| dvD vol. | NP12 | NP13 | NP17 | NP28 | NP29 | NP10 | NP11 | NP14 | NP25 | NP26 | NP27 | NP31 | NP34 |
| pre | 17761920 | 19645760 | 5113280 | 67549120 | 9150080 | 19376640 | 16685440 | 5651520 | 9688320 | 11033920 | 11033920 | 13725120 | 15070720 |
| 1d | 12648640 | 8073600 | 6189760 | 4844160 | 11841280 | 17492800 | 31217920 | 6458880 | 35254720 | 11033920 | 30410560 | 2960320 | 6189760 |
| 4d | 9150080 | 8073600 | 27719360 | 9419200 | 14801600 | 8611840 | 6997120 | 17492800 | 67818240 | 32832640 | 28526720 | 47634240 | 43059200 |
| 8d | 21529600 | 8880960 | 10495680 | 44943040 | 18300160 | 64588800 | 89886080 | 1.05E+08 | 1.61E+08 | 87194880 | 43866560 | 77506560 | 1.35E+08 |
| 16d | 28257600 | 22067840 | 5651520 | 29334080 | 13186880 | 73738880 | 71585920 | 1.17E+08 | 2.68E+08 | 1.56E+08 | 67818240 | 1.56E+08 | 1.69E+08 |
| 31d | 15608960 | 34716480 | 18569280 | 32294400 | 8611840 | 51401920 | 1.33E+08 | 1.25E+08 | 1.86E+08 | 1.75E+08 | 1.03E+08 | 1.62E+08 | 2.48E+08 |
|  |  |  |  |  |  |  |  |  |  |  |  |  |  |

**Figure 6 - source data 1: Summary of dorsoventral diffusivity metrics.** Quantitative values of dorsoventral diffusivity (dvD) measurements are listed for individual mice (saline-injected: N12, NP13, NP17, NP28, NP29; kainate-injected: NP10, NP11, NP14, NP25, NP26, NP27, NP31, NP34) and longitudinal time points (pre, 1d, 4d, 8d, 16d, 31d following injection).
