# Supplementary material for: Early tissue damage and microstructural reorganization predict disease severity in experimental epilepsy
Source: eLife. 2017 Jul 26;6:e25742. doi: 10.7554/eLife.25742 (PMC5529108; doi:10.7554/eLife.25742)
Supplement: Supplementary file 1. — The table displays all results statistical tests performed (right column) for each parameter (left column). The reference to the corresponding figure is given in the middle column. CI, confidence interval; n, number of animals; n*, number of sections; n°, number of recordings. DOI: http://dx.doi.org/10.7554/eLife.25742.024 [file elife-25742-supp1.docx]

| **Parameter** | **Reference** | **Statistical test and summary of results** | | | | | | | |  |
| --- | --- | --- | --- | --- | --- | --- | --- | --- | --- | --- |
|  |  |  | |  | |  |  |  |  |  |
| **mean GCD vol.** | **Fig. 1C** | One-way ANOVA | |  | |  |  |  |  |  |
|  |  | P value | | < 0.0001 | |  |  |  |  |  |
|  |  | Number of groups | | 8 | |  |  |  |  |  |
|  |  | F value | | 25.99 | |  |  |  |  |  |
|  |  |  | |  | |  |  |  |  |  |
|  |  | Bonferroni's Multiple Comparison Test | | | |  |  |  |  |  |
|  |  | Groups (n*) | | Mean Diff. | | 95% CI | P values |  |  |  |
|  |  | NP27 (14) vs NP10 (12) | | -29980 | | -294200 to 234200 | > 0.05 |  |  |  |
|  |  | NP27 (14) vs NP11 (27) | | -190300 | | -411400 to 30890 | > 0.05 |  |  |  |
|  |  | NP27 (14) vs NP26 (37) | | -394600 | | -605300 to -183900 | < 0.001 |  |  |  |
|  |  | NP27 (14) vs NP31 (37) | | -391700 | | -602400 to -181000 | < 0.001 |  |  |  |
|  |  | NP27 (14) vs NP14 (44) | | -421000 | | -627100 to -215000 | < 0.001 |  |  |  |
|  |  | NP27 (14) vs NP34 (45) | | -507600 | | -713100 to -302100 | < 0.001 |  |  |  |
|  |  | NP27 (14) vs NP25 (41) | | -652400 | | -860300 to -444500 | < 0.001 |  |  |  |
|  |  | NP10 (12) vs NP11 (27) | | -160300 | | -393300 to 72690 | > 0.05 |  |  |  |
|  |  | NP10 (12) vs NP26 (37) | | -364600 | | -587700 to -141500 | < 0.001 |  |  |  |
|  |  | NP10 (12) vs NP31 (37) | | -361800 | | -584800 to -138700 | < 0.001 |  |  |  |
|  |  | NP10 (12) vs NP14 (44) | | -391100 | | -609700 to -172400 | < 0.001 |  |  |  |
|  |  | NP10 (12) vs NP34 (45) | | -477700 | | -695800 to -259500 | < 0.001 |  |  |  |
|  |  | NP10 (12) vs NP25 (41) | | -622400 | | -842800 to -402000 | < 0.001 |  |  |  |
|  |  | NP11 (27) vs NP26 (37) | | -204300 | | -374300 to -34320 | < 0.01 |  |  |  |
|  |  | NP11 (27) vs NP31 (37) | | -201500 | | -371400 to -31490 | < 0.01 |  |  |  |
|  |  | NP11 (27) vs NP14 (44) | | -230800 | | -394900 to -66600 | < 0.001 |  |  |  |
|  |  | NP11 (27) vs NP34 (45) | | -317400 | | -480800 to -153900 | < 0.001 |  |  |  |
|  |  | NP11 (27) vs NP25 (41) | | -462100 | | -628600 to -295700 | < 0.001 |  |  |  |
|  |  | NP26 (37) vs NP31 (37) | | 2824 | | -153300 to 158900 | > 0.05 |  |  |  |
|  |  | NP26 (37) vs NP14 (44) | | -26480 | | -176300 to 123300 | > 0.05 |  |  |  |
|  |  | NP26 (37) vs NP34 (45) | | -113100 | | -262100 to 35940 | > 0.05 |  |  |  |
|  |  | NP26 (37) vs NP25 (41) | | -257800 | | -410100 to -105600 | < 0.001 |  |  |  |
|  |  | NP31 (37) vs NP14 (44) | | -29300 | | -179100 to 120500 | > 0.05 |  |  |  |
|  |  | NP31 (37) vs NP34 (45) | | -115900 | | -264900 to 33110 | > 0.05 |  |  |  |
|  |  | NP31 (37) vs NP25 (41) | | -260700 | | -412900 to -108400 | < 0.001 |  |  |  |
|  |  | NP14 (44) vs NP34 (45) | | -86610 | | -229000 to 55760 | > 0.05 |  |  |  |
|  |  | NP14 (44) vs NP25 (41) | | -231400 | | -377100 to -85590 | < 0.001 |  |  |  |
|  |  | NP34 (45) vs NP25 (41) | | -144700 | | -289700 to 232.9 | > 0.05 |  |  |  |
|  |  |  | |  | |  |  |  |  |  |
|  |  |  | |  | |  |  |  |  |  |
| **Seiz.-like freq.** | **Fig1, S1H** | One-way ANOVA | |  | |  |  |  |  |  |
|  |  | P value | | < 0.0001 | |  |  |  |  |  |
|  |  | Number of groups | | 5 | |  |  |  |  |  |
|  |  | F value | | 9.614 | |  |  |  |  |  |
|  |  | R² values | | 0.5618 | |  |  |  |  |  |
|  |  |  | |  | |  |  |  |  |  |
|  |  | Bonferroni's Multiple Comparison Test | |  | |  |  |  |  |  |
|  |  | Groups (n*) | | Mean Diff. | | 95% CI | P values |  |  |  |
|  |  | 1d vs 0.5w | | 0.01208 | | -0.1663 to 0.1905 | > 0.05 |  |  |  |
|  |  | 1d vs 1w | | -0.0225 | | -0.1877 to 0.1427 | > 0.05 |  |  |  |
|  |  | 1d vs 2w | | -0.2263 | | -0.3914 to -0.06109 | < 0.01 |  |  |  |
|  |  | 1d vs 2w | | -0.2603 | | -0.4486 to -0.07194 | < 0.01 |  |  |  |
|  |  | 0.5w vs 1w | | -0.03458 | | -0.2130 to 0.1438 | > 0.05 |  |  |  |
|  |  | 0.5w vs 2w | | -0.2383 | | -0.4167 to -0.05994 | < 0.01 |  |  |  |
|  |  | 0.5w vs 3w | | -0.2723 | | -0.4724 to -0.07232 | < 0.01 |  |  |  |
|  |  | 1w vs 2w | | -0.2038 | | -0.3689 to -0.03859 | < 0.01 |  |  |  |
|  |  | 1w vs 3w | | -0.2378 | | -0.4261 to -0.04944 | < 0.01 |  |  |  |
|  |  | 2w vs 3w | | -0.03400 | | -0.2223 to 0.1543 | > 0.05 |  |  |  |
|  |  |  | |  | |  |  |  |  |  |
| **total GCL vol.** | **Fig. 1F** | Pearson's correlation | |  | |  |  |  |  |  |
| **vs.** |  | Time point | | Number of X values | | Slope | 95% CI | R² value | P value |  |
| **microgliosis** |  | 36d | | 8 | | 148300 ± 40170 | 49960 to 246600 | 0.6942 | 0.0102 |  |
|  |  |  | |  | |  |  |  |  |  |
| **total GCL vol.** | **Fig. 1H** | Unpaired Student's t test. two-tailed | | | |  |  |  |  |  |
|  |  | Non-epileptic (n = 6) [*10^-5^] | | Epileptic (n = 7) [*10^5^] | | Difference [*10^5^] | 95% CI [*10^5^] | R² value | P value |  |
|  |  | 4118 ± 103.1 | | 12450 ± 1931 | | -8330 ± 2102 | -12960 to -3704 | 0.5881 | 0.0022 |  |
|  |  |  | |  | |  |  |  |  |  |
| **microgliosis** | **Fig. 1I** | Unpaired Student's t test. two-tailed | | | |  |  |  |  |  |
|  |  | Non-epileptic (n = 6) | | Epileptic (n = 7) | | Difference | 95% CI | R² value | P value |  |
|  |  | 70.17 ± 11.06 | | 6474 ± 843.9 | | -6404 ± 917.5 | -8423 to -4384 | 0.8158 | < 0.0001 |  |
|  |  |  | |  | |  |  |  |  |  |
| **T2 (CA1)** | **Fig. 2G** | Two-way ANOVA | |  | |  |  |  |  |  |
|  |  | Source of Variation | | Interaction | | Time | Treatment | Subjects |  |  |
|  |  | % of total variation | | 32.19 | | 33.50 | 4.60 | 9.1387 |  |  |
|  |  | P value | | < 0.0001 | | < 0.0001 | 0.0384 | 0.0034 |  |  |
|  |  | F value | | 23.25 | | 24.19 | 5.532 | 3.000 |  |  |
|  |  |  | |  | |  |  |  |  |  |
|  |  | Bonferroni posttests | |  | |  |  |  |  |  |
|  |  | Time point | | Non-epileptic (n = 6) | | Epileptic (n = 7) | Difference | 95% CI | P value |  |
|  |  | pre | | 10.68 | | 10.61 | -0.06228 | -1.090 to 0.9651 | > 0.05 |  |
|  |  | 1d | | 10.59 | | 13.45 | 2.856 | 1.829 to 3.883 | < 0.001 |  |
|  |  | 4d | | 10.33 | | 10.94 | 0.6162 | -0.4112 to 1.644 | > 0.05 |  |
|  |  | 8d | | 10.51 | | 10.53 | 0.01859 | -1.009 to 1.046 | > 0.05 |  |
|  |  | 16d | | 10.68 | | 10.12 | -0.5567 | -1.584 to 0.4706 | > 0.05 |  |
|  |  | 31d | | 10.42 | | 10.14 | -0.2842 | -1.312 to 0.7431 | > 0.05 |  |
|  |  |  | |  | |  |  |  |  |  |
| **T2 (CA1)** | **Fig. 2H** | Pearson's correlation. corrected for multiple comparisons | | | | |  |  |  |  |
| **vs.** |  | Time point | | Number of X values | | Slope [*10^-4^] | 95% CI [*10^-4^] | R² value | P value |  |
| **microgliosis** |  | pre | | 13 | | -0.2824 ± 0.3448 | -1.041 to 0.4764 | 0.05750 | 2.5806 |  |
|  |  | 1d | | 13 | | 4.102 ± 0.4531 | 3.104 to 5.099 | 0.8817 | < 0.0006 |  |
|  |  | 4d | | 13 | | 1.325 ± 0.5004 | 0.2235 to 2.426 | 0.3892 | 0.1362 |  |
|  |  | 8d | | 13 | | 0.09008 ± 0.2990 | -0.5680 to 0.7481 | 0.008186 | 4.6128 |  |
|  |  | 16d | | 13 | | -0.7815 ± 0.3302 | -1.508 to -0.05484 | 0.3375 | 0.2238 |  |
|  |  | 31d | | 13 | | -0.3466 ± 0.3861 | -1.196 to 0.5032 | 0.06824 | 2.3316 |  |
|  |  |  | |  | |  |  |  |  |  |
| **T2 (DG)** | **Fig. 2I** | Two-way ANOVA | |  | |  |  |  |  |  |
|  |  | Source of Variation | | Interaction | | Time | Treatment | Subjects |  |  |
|  |  | % of total variation | | 10.47 | | 16.78 | 25.84 | 18.4162 |  |  |
|  |  | P value | | 0.0022 | | < 0.0001 | 0.0024 | 0.0011 |  |  |
|  |  | F value | | 4.320 | | 6.919 | 15.44 | 3.452 |  |  |
|  |  |  | |  | |  |  |  |  |  |
|  |  | Bonferroni posttests | |  | |  |  |  |  |  |
|  |  | Time point | | Non-epileptic (n = 6) | | Epileptic (n = 7) | Difference | 95% CI | P value |  |
|  |  | pre | | 10.85 | | 10.76 | -0.09225 | -0.9558 to 0.7713 | > 0.05 |  |
|  |  | 1d | | 10.71 | | 11.85 | 1.139 | 0.2758 to 2.003 | < 0.001 |  |
|  |  | 4d | | 10.61 | | 10.94 | 0.3374 | -0.5262 to 1.201 | > 0.05 |  |
|  |  | 8d | | 10.81 | | 11.65 | 0.8449 | -0.01862 to 1.709 | < 0.05 |  |
|  |  | 16d | | 11.09 | | 11.80 | 0.7020 | -0.1616 to 1.566 | > 0.05 |  |
|  |  | 31d | | 10.86 | | 11.72 | 0.8605 | -0.003020 to 1.724 | < 0.05 |  |
|  |  |  | |  | |  |  |  |  |  |
| **T2 (DG)** | **Fig. 2J** | Pearson's correlation. corrected for multiple comparisons | | | | |  |  |  |  |
| **vs.** |  | Time point | | Number of X values | | Slope [*10^-9^] | 95% CI [*10^-9^] | R² value | P value |  |
| **total GCL vol.** |  | pre | | 13 | | -0.255 ± 0.192 | -0.678 to 0.168 | 0.1378 | 1.2708 |  |
|  |  | 1d | | 13 | | 1.021 ± 0.224 | 0.528 to 1.514 | 0.6537 | 0.0048 |  |
|  |  | 4d | | 13 | | 0.26 ± 0.217 | -0.218 to 0.738 | 0.1154 | 1.5372 |  |
|  |  | 8d | | 13 | | 0.717 ± 0.209 | 0.257 to 1.178 | 0.5167 | 0.0336 |  |
|  |  | 16d | | 13 | | 0.752 ± 0.22 | 0.268 to 1.235 | 0.5158 | 0.0342 |  |
|  |  | 31d | | 13 | | 0.968 ± 0.255 | 0.406 to 1.529 | 0.5668 | 0.018 |  |
|  |  |  | |  | |  |  |  |  |  |
| **NAA conc.** | **Fig. 3C** | Two-way ANOVA | |  | |  |  |  |  |  |
|  |  | Source of Variation | | Interaction | | Time | Treatment | Subjects |  |  |
|  |  | % of total variation | | 8.28 | | 17.15 | 57.47 | 11.2618 |  |  |
|  |  | P value | | < 0.0001 | | < 0.0001 | < 0.0001 | < 0.0001 |  |  |
|  |  | F value | | 22.97 | | 47.59 | 56.13 | 14.21 |  |  |
|  |  |  | |  | |  |  |  |  |  |
|  |  | Bonferroni posttests | |  | |  |  |  |  |  |
|  |  | Time point | | Non-epileptic (n = 6) | | Epileptic (n = 7) | Difference | 95% CI | P value |  |
|  |  | pre | | 82.71 | | 77.86 | -4.853 | -15.68 to 5.972 | > 0.05 |  |
|  |  | 1d | | 77.89 | | 42.58 | -35.31 | -46.13 to -24.48 | < 0.001 |  |
|  |  | 4d | | 74.12 | | 44.11 | -30.01 | -40.84 to -19.19 | < 0.001 |  |
|  |  | 8d | | 76.87 | | 49.47 | -27.40 | -38.23 to -16.58 | < 0.001 |  |
|  |  | 16d | | 82.00 | | 51.86 | -30.13 | -40.96 to -19.31 | < 0.001 |  |
|  |  | 31d | | 79.98 | | 53.84 | -26.14 | -36.97 to -15.32 | < 0.001 |  |
|  |  |  | |  | |  |  |  |  |  |
| **NAA conc.** | **Fig. 3D** | Pearson's correlation. corrected for multiple comparisons | | | | |  |  |  |  |
| **vs.** |  | Time point | | Number of X values | | Slope [*10^-3^] | 95% CI [*10^-3^] | R² value | P value |  |
| **microgliosis** |  | pre | | 13 | | -0.7030 ± 0.3850 | -1.550 to 0.1444 | 0.2326 | 0.5706 |  |
|  |  | 1d | | 13 | | -5.004 ± 0.5125 | -6.132 to -3.876 | 0.8966 | < 0.0006 |  |
|  |  | 4d | | 13 | | -3.895 ± 0.7214 | -5.482 to -2.307 | 0.7260 | 0.0012 |  |
|  |  | 8d | | 13 | | -3.767 ± 0.7252 | -5.363 to -2.170 | 0.7103 | 0.0018 |  |
|  |  | 16d | | 13 | | -4.267 ± 0.7096 | -5.829 to -2.705 | 0.7668 | < 0.0006 |  |
|  |  | 31d | | 13 | | -3.625 ± 0.5019 | -4.730 to -2.521 | 0.8259 | < 0.0006 |  |
|  |  |  | |  | |  |  |  |  |  |
| **Glu conc.** | **Fig. 3E** | Two-way ANOVA | |  | |  |  |  |  |  |
|  |  | Source of Variation | | Interaction | | Time | Treatment | Subjects |  |  |
|  |  | % of total variation | | 8.92 | | 14.02 | 49.95 | 15.9664 |  |  |
|  |  | P value | | < 0.0001 | | < 0.0001 | 0.0001 | < 0.0001 |  |  |
|  |  | F value | | 10.20 | | 16.04 | 34.41 | 8.302 |  |  |
|  |  |  | |  | |  |  |  |  |  |
|  |  | Bonferroni posttests | |  | |  |  |  |  |  |
|  |  | Time point | | Non-epileptic (n = 6) | | Epileptic (n = 7) | Difference | 95% CI | P value |  |
|  |  | pre | | 103.2 | | 99.59 | -3.625 | -18.00 to 10.75 | > 0.05 |  |
|  |  | 1d | | 102.1 | | 66.23 | -35.86 | -50.23 to -21.48 | < 0.001 |  |
|  |  | 4d | | 93.15 | | 71.97 | -21.19 | -35.56 to -6.814 | < 0.01 |  |
|  |  | 8d | | 97.49 | | 71.32 | -26.17 | -40.55 to -11.80 | < 0.001 |  |
|  |  | 16d | | 104.4 | | 73.28 | -31.13 | -45.51 to -16.76 | < 0.001 |  |
|  |  | 31d | | 98.48 | | 69.53 | -28.96 | -43.33 to -14.58 | < 0.001 |  |
|  |  |  | |  | |  |  |  |  |  |
| **Glu conc.** | **Fig. 3F** | Pearson's correlation. corrected for multiple comparisons | | | | |  |  |  |  |
| **vs.** |  | Time point | | Number of X values | | Slope [*10^-3^] | 95% CI [*10^-3^] | R² value | P value |  |
| **microgliosis** |  | pre | | 13 | | -0.5529 ± 0.2562 | -1.117 to 0.01101 | 0.2974 | 0.3234 |  |
|  |  | 1d | | 13 | | -5.182 ± 0.4336 | -6.136 to -4.228 | 0.9285 | < 0.0006 |  |
|  |  | 4d | | 13 | | -2.698 ± 0.8680 | -4.609 to -0.7876 | 0.4676 | 0.06 |  |
|  |  | 8d | | 13 | | -4.067 ± 0.8779 | -5.999 to -2.135 | 0.6612 | 0.0042 |  |
|  |  | 16d | | 13 | | -4.737 ± 0.6983 | -6.274 to -3.200 | 0.8071 | < 0.0006 |  |
|  |  | 31d | | 13 | | -4.184 ± 0.6858 | -5.693 to -2.674 | 0.7719 | < 0.0006 |  |
|  |  |  | |  | |  |  |  |  |  |
| **GABA conc.** | **Fig. 3G** | Two-way ANOVA | |  | |  |  |  |  |  |
|  |  | Source of Variation | | Interaction | | Time | Treatment | Subjects |  |  |
|  |  | % of total variation | | 21.05 | | 20.06 | 13.85 | 15.5166 |  |  |
|  |  | P value | | < 0.0001 | | < 0.0001 | 0.0095 | 0.0042 |  |  |
|  |  | F value | | 8.721 | | 8.312 | 9.818 | 2.922 |  |  |
|  |  |  | |  | |  |  |  |  |  |
|  |  | Bonferroni posttests | |  | |  |  |  |  |  |
|  |  | Time point | | Non-epileptic (n = 6) | | Epileptic (n = 7) | Difference | 95% CI | P value |  |
|  |  | pre | | 41.28 | | 40.79 | -0.4867 | -9.519 to 8.545 | > 0.05 |  |
|  |  | 1d | | 44.14 | | 26.76 | -17.38 | -26.41 to -8.350 | < 0.001 |  |
|  |  | 4d | | 39.28 | | 28.29 | -10.99 | -20.02 to -1.955 | < 0.05 |  |
|  |  | 8d | | 44.39 | | 33.01 | -11.38 | -20.41 to -2.346 | < 0.05 |  |
|  |  | 16d | | 44.82 | | 41.05 | -3.761 | -12.79 to 5.271 | > 0.05 |  |
|  |  | 31d | | 41.26 | | 47.34 | 6.081 | -2.951 to 15.11 | > 0.05 |  |
|  |  |  | |  | |  |  |  |  |  |
| **GABA conc.** | **Fig. 3H** | Pearson's correlation. corrected for multiple comparisons | | | | |  |  |  |  |
| **vs.** |  | Time point | | Number of X values | | Slope [*10^-3^] | 95% CI [*10^-3^] | R² value | P value |  |
| **microgliosis** |  | pre | | 13 | | -0.1298 ± 0.3629 | -0.9285 to 0.6689 | 0.01150 | 4.3644 |  |
|  |  | 1d | | 13 | | -2.534 ± 0.4157 | -3.449 to -1.619 | 0.7716 | < 0.0006 |  |
|  |  | 4d | | 13 | | -1.737 ± 0.3085 | -2.417 to -1.058 | 0.7425 | 0.0012 |  |
|  |  | 8d | | 13 | | -1.470 ± 0.4936 | -2.556 to -0.3836 | 0.4464 | 0.0756 |  |
|  |  | 16d | | 13 | | -0.08544 ± 0.6441 | -1.503 to 1.332 | 0.001597 | 5.3814 |  |
|  |  | 31d | | 13 | | 1.202 ± 0.4017 | 0.3178 to 2.086 | 0.4487 | 0.0738 |  |
|  |  |  | |  | |  |  |  |  |  |
|  |  |  | |  | |  |  |  |  |  |
| **Lac conc.** | **Fig. 3I** | Two-way ANOVA | |  | |  |  |  |  |  |
|  |  | Source of Variation | | Interaction | | Time | Treatment | Subjects |  |  |
|  |  | % of total variation | | 9.83 | | 13.94 | 22.08 | 20.2573 |  |  |
|  |  | P value | | 0.0099 | | 0.0011 | 0.0053 | 0.0023 |  |  |
|  |  | F value | | 3.374 | | 4.785 | 11.99 | 3.161 |  |  |
|  |  |  | |  | |  |  |  |  |  |
|  |  | Bonferroni posttests | |  | |  |  |  |  |  |
|  |  | Time point | | Non-epileptic (n = 6) | | Epileptic (n = 7) | Difference | 95% CI | P value |  |
|  |  | pre | | 18.89 | | 19.82 | 0.9274 | -35.47 to 37.32 | > 0.05 |  |
|  |  | 1d | | 25.30 | | 77.72 | 52.41 | 16.02 to 88.81 | < 0.01 |  |
|  |  | 4d | | 18.27 | | 69.65 | 51.38 | 14.98 to 87.77 | < 0.01 |  |
|  |  | 8d | | 12.24 | | 45.14 | 32.90 | -3.502 to 69.29 | > 0.05 |  |
|  |  | 16d | | 15.13 | | 40.37 | 25.24 | -11.16 to 61.64 | > 0.05 |  |
|  |  | 31d | | 18.22 | | 28.39 | 10.17 | -26.23 to 46.57 | > 0.05 |  |
|  |  |  | |  | |  |  |  |  |  |
| **Lac conc.** | **Figure 3J** | Pearson's correlation. corrected for multiple comparisons | | | | |  |  |  |  |
| **vs.** |  | Time point | | Number of X values | | Slope [*10^-3^] | 95% CI [*10^-3^] | R² value | P value |  |
| **microgliosis** |  | pre | | 13 | | 0.3700 ± 0.4445 | -0.6083 to 1.348 | 0.05927 | 2.5368 |  |
|  |  | 1d | | 13 | | 9.272 ± 2.768 | 3.179 to 15.37 | 0.5049 | 0.039 |  |
|  |  | 4d | | 13 | | 7.883 ± 2.225 | 2.986 to 12.78 | 0.5330 | 0.0276 |  |
|  |  | 8d | | 13 | | 4.369 ± 1.133 | 1.875 to 6.862 | 0.5748 | 0.0162 |  |
|  |  | 16d | | 13 | | 4.249 ± 1.130 | 1.763 to 6.736 | 0.5626 | 0.0186 |  |
|  |  | 31d | | 13 | | 2.239 ± 1.315 | -0.6567 to 5.134 | 0.2084 | 0.7014 |  |
|  |  |  | |  | |  |  |  |  |  |
| **Myoi conc.** | **Fig. 3K** | Two-way ANOVA | |  | |  |  |  |  |  |
|  |  | Source of Variation | | Interaction | | Time | Treatment | Subjects |  |  |
|  |  | % of total variation | | 16.34 | | 15.61 | 6.73 | 46.5296 |  |  |
|  |  | P value | | < 0.0001 | | < 0.0001 | 0.2332 | < 0.0001 |  |  |
|  |  | F value | | 14.78 | | 14.12 | 1.592 | 19.13 |  |  |
|  |  |  | |  | |  |  |  |  |  |
|  |  | Bonferroni posttests | |  | |  |  |  |  |  |
|  |  | Time point | | Non-epileptic (n = 6) | | Epileptic (n = 7) | Difference | 95% CI | P value |  |
|  |  | pre | | 66.17 | | 68.22 | 2.058 | -18.30 to 22.41 | > 0.05 |  |
|  |  | 1d | | 64.54 | | 49.59 | -14.95 | -35.31 to 5.401 | > 0.05 |  |
|  |  | 4d | | 65.93 | | 79.22 | 13.30 | -7.056 to 33.65 | > 0.05 |  |
|  |  | 8d | | 63.56 | | 90.15 | 26.59 | 6.234 to 46.94 | < 0.01 |  |
|  |  | 16d | | 65.47 | | 82.96 | 17.49 | -2.864 to 37.84 | > 0.05 |  |
|  |  | 31d | | 66.94 | | 72.89 | 5.953 | -14.40 to 26.31 | > 0.05 |  |
|  |  |  | |  | |  |  |  |  |  |
| **Myoi conc.** | **Figure 3L** | Pearson's correlation. corrected for multiple comparisons | | | | |  |  |  |  |
| **vs.** |  | Time point | | Number of X values | | Slope [*10^-3^] | 95% CI [*10^-3^] | R² value | P value |  |
| **microgliosis** |  | pre | | 13 | | -0.02934 ± 0.7553 | -1.692 to 1.633 | 0.0001372 | 5.8182 |  |
|  |  | 1d | | 13 | | -2.315 ± 0.8596 | -4.207 to -0.4230 | 0.3974 | 0.1254 |  |
|  |  | 4d | | 13 | | 1.364 ± 1.365 | -1.640 to 4.368 | 0.08329 | 2.0334 |  |
|  |  | 8d | | 13 | | 3.330 ± 1.236 | 0.6086 to 6.051 | 0.3974 | 0.1254 |  |
|  |  | 16d | | 13 | | 1.744 ± 1.368 | -1.267 to 4.755 | 0.1287 | 1.3716 |  |
|  |  | 31d | | 13 | | 0.8484 ± 0.8556 | -1.035 to 2.732 | 0.08205 | 2.0562 |  |
|  |  |  | |  | |  |  |  |  |  |
|  |  |  | |  | |  |  |  |  |  |
| **width of GCL** | **Fig. 4B** | One-way ANOVA | |  | |  |  |  |  |  |
|  |  | P value | | < 0.0001 | |  |  |  |  |  |
|  |  | Number of groups | | 6 | |  |  |  |  |  |
|  |  | F value | | 29.58 | |  |  |  |  |  |
|  |  |  | |  | |  |  |  |  |  |
|  |  | Dunnett's Multiple Comparison Test | | | |  |  |  |  |  |
|  |  | Groups (n) | | Mean Diff. | | 95% CI | P values |  |  |  |
|  |  | saline (7) vs KA 1d (3) | | 3.562 | | -28.71 to 35.84 | > 0.05 |  |  |  |
|  |  | saline (7) vs KA 4d (5) | | -13.38 | | -40.77 to 14.01 | > 0.05 |  |  |  |
|  |  | saline (7) vs KA 7d (6) | | -24.99 | | -51.01 to 1.036 | > 0.05 |  |  |  |
|  |  | saline (7) vs KA 14d (3) | | -64.83 | | -97.11 to -32.55 | < 0.001 |  |  |  |
|  |  | saline (7) vs KA 21d (6) | | -100.8 | | -126.8 to -74.75 | < 0.001 |  |  |  |
|  |  |  | |  | |  |  |  |  |  |
| **dendrite diam.** | **Fig. 4D (1)** | One-way ANOVA | |  | |  |  |  |  |  |
|  |  | P value | | < 0.0001 | |  |  |  |  |  |
|  |  | Number of groups | | 6 | |  |  |  |  |  |
|  |  | F value | | 32.00 | |  |  |  |  |  |
|  |  |  | |  | |  |  |  |  |  |
|  |  | Dunnett's Multiple Comparison Test | | | |  |  |  |  |  |
|  |  | Groups (n) | | Mean Diff. | | 95% CI | P values |  |  |  |
|  |  | saline (5) vs KA 1d (3) | | 0.2052 | | -0.1249 to 0.5353 | > 0.05 |  |  |  |
|  |  | saline (5) vs KA 4d (5) | | -0.1806 | | -0.4665 to 0.1053 | > 0.05 |  |  |  |
|  |  | saline (5) vs KA 7d (4) | | -0.3265 | | -0.6298 to -0.02331 | < 0.05 |  |  |  |
|  |  | saline (5) vs KA 14d (3) | | -0.7471 | | -1.077 to -0.4170 | < 0.001 |  |  |  |
|  |  | saline (5) vs KA 21d (5) | | -1.010 | | -1.296 to -0.7239 | < 0.001 |  |  |  |
|  |  |  | |  | |  |  |  |  |  |
| **axon diam.** | **Fig. 4D (2)** | One-way ANOVA | |  | |  |  |  |  |  |
|  |  | P value | | < 0.0001 | |  |  |  |  |  |
|  |  | Number of groups | | 6 | |  |  |  |  |  |
|  |  | F value | | 43.86 | |  |  |  |  |  |
|  |  |  | |  | |  |  |  |  |  |
|  |  | Dunnett's Multiple Comparison Test | | | |  |  |  |  |  |
|  |  | Groups (n) | | Mean Diff. | | 95% CI | P values |  |  |  |
|  |  | saline (5) vs KA 1d (3) | | -0.001400 | | -0.2446 to 0.2418 | > 0.05 |  |  |  |
|  |  | saline (5) vs KA 4d (5) | | -0.02680 | | -0.2374 to 0.1838 | > 0.05 |  |  |  |
|  |  | saline (5) vs KA 7d (4) | | -0.1019 | | -0.3253 to 0.1215 | > 0.05 |  |  |  |
|  |  | saline (5) vs KA 14d (3) | | -0.4477 | | -0.6909 to -0.2045 | < 0.001 |  |  |  |
|  |  | saline (5) vs KA 21d (5) | | -0.9242 | | -1.135 to -0.7136 | < 0.001 |  |  |  |
|  |  |  | |  | |  |  |  |  |  |
| **ZnT-3 density** | **Fig. 4F** | One-way ANOVA | |  | |  |  |  |  |  |
|  |  | P value | | < 0.0001 | |  |  |  |  |  |
|  |  | Number of groups | | 6 | |  |  |  |  |  |
|  |  | F value | | 7.740 | |  |  |  |  |  |
|  |  | R² value | | 0.5633 | |  |  |  |  |  |
|  |  |  | |  | |  |  |  |  |  |
|  |  | Dunnett's Multiple Comparison Test | | | |  |  |  |  |  |
|  |  | Groups (n) | | Mean Diff. | | 95% CI | P values |  |  |  |
|  |  | saline (8) vs KA 1d (4) | | -0.8536 | | -7.426 to 5.719 | > 0.05 |  |  |  |
|  |  | saline (8) vs KA 4d (5) | | -1.841 | | -7.959 to 4.278 | > 0.05 |  |  |  |
|  |  | saline (8) vs KA 7d (5) | | -5.620 | | -11.74 to 0.4991 | > 0.05 |  |  |  |
|  |  | saline (8) vs KA 14d (5) | | -7.628 | | -13.75 to -1.510 | < 0.05 |  |  |  |
|  |  | saline (8) vs KA 21d (9) | | -10.55 | | -15.77 to -5.337 | < 0.001 |  |  |  |
|  |  |  | |  | |  |  |  |  |  |
| **mean GFAP vol.** | **Fig. 4H** | One-way ANOVA | |  | |  |  |  |  |  |
|  |  | P value | | < 0.0001 | |  |  |  |  |  |
|  |  | Number of groups | | 6 | |  |  |  |  |  |
|  |  | F value | | 22.88 | |  |  |  |  |  |
|  |  | R² value | | 0.8449 | |  |  |  |  |  |
|  |  |  | |  | |  |  |  |  |  |
|  |  | Dunnett's Multiple Comparison Test | | | |  |  |  |  |  |
|  |  | Groups (n) | | Mean Diff. | | 95% CI | P values |  |  |  |
|  |  | saline (5) vs KA 1d (4) | | -1.500 | | -9.471 to 6.471 | > 0.05 |  |  |  |
|  |  | saline (5) vs KA 4d (5) | | -12.62 | | -20.14 to -5.106 | < 0.001 |  |  |  |
|  |  | saline (5) vs KA 7d (4) | | -14.13 | | -22.10 to -6.155 | < 0.001 |  |  |  |
|  |  | saline (5) vs KA 14d (4) | | -22.79 | | -30.76 to -14.82 | < 0.001 |  |  |  |
|  |  | saline (5) vs KA 21d (5) | | -22.28 | | -29.80 to -14.77 | < 0.001 |  |  |  |
|  |  |  | |  | |  |  |  |  |  |
| **total GFAP vol.** | **Fig. 4I** | One-way ANOVA | |  | |  |  |  |  |  |
|  |  | P value | | < 0.0001 | |  |  |  |  |  |
|  |  | Number of groups | | 6 | |  |  |  |  |  |
|  |  | F value | | 10.02 | |  |  |  |  |  |
|  |  | R² value | | 0.7046 | |  |  |  |  |  |
|  |  |  | |  | |  |  |  |  |  |
|  |  | Dunnett's Multiple Comparison Test | | | |  |  |  |  |  |
|  |  | Groups (n) | | Mean Diff. | | 95% CI | P values |  |  |  |
|  |  | saline (5) vs KA 1d (4) | | -2395 | | -6185 to 1394 | > 0.05 |  |  |  |
|  |  | saline (5) vs KA 4d (5) | | -6664 | | -10240 to -3091 | < 0.001 |  |  |  |
|  |  | saline (5) vs KA 7d (4) | | -5736 | | -9525 to -1946 | < 0.01 |  |  |  |
|  |  | saline (5) vs KA 14d (4) | | -7628 | | -11420 to -3838 | < 0.001 |  |  |  |
|  |  | saline (5) vs KA 21d (5) | | -6997 | | -10570 to -3425 | < 0.001 |  |  |  |
|  |  |  | |  | |  |  |  |  |  |
| **mean GFAP dens.** | **Fig. 4L** | One-way ANOVA | |  | |  |  |  |  |  |
|  |  | P value | | < 0.0001 | |  |  |  |  |  |
|  |  | Number of groups | | 3 | |  |  |  |  |  |
|  |  | F value | | 56.52 | |  |  |  |  |  |
|  |  | R² value | | 0.6244 | |  |  |  |  |  |
|  |  |  | |  | |  |  |  |  |  |
|  |  | Bonferroni's Multiple Comparison Test | |  | |  |  |  |  |  |
|  |  | Groups (n*) | | | | Mean Diff. | 95% CI | P values |  |  |
|  |  | no GCD (51) vs wk. GCD (6) | | -5.471 | | -8.623 to -2.318 | < 0.001 |  |  |  |
|  |  | no GCD (51) vs str. GCD (14) | | -9.222 | | -11.43 to -7.018 | < 0.001 |  |  |  |
|  |  | Wk. GCD (6) vs str. GCD (14) | | -3.751 | | -7.315 to -0.1874 | < 0.05 |  |  |  |
|  |  |  | |  | |  |  |  |  |  |
| **integr. GFAP dens.** | **Fig. 1F** | Pearson's correlation | |  | |  |  |  |  |  |
| **vs.** |  | Time point | | Number of X values | | Slope | 95% CI | R² value | P value |  |
| **GCL area** |  | 36d | | 20 | | 35.32 ± 2.862 | 29.31 to 41.33 | 0.8943 | < 0.0001 |  |
|  |  |  | |  | |  |  |  |  |  |
| **MD (DG)** | **Fig. 5I** | Two-way ANOVA | |  | |  |  |  |  |  |
|  |  | Source of Variation | | Interaction | | Time | Treatment | Subjects |  |  |
|  |  | % of total variation | | 12.53 | | 36.06 | 17.08 | 10.2962 |  |  |
|  |  | P value | | < 0.0001 | | < 0.0001 | 0.0013 | 0.0136 |  |  |
|  |  | F value | | 6.619 | | 19.05 | 18.25 | 2.472 |  |  |
|  |  |  | |  | |  |  |  |  |  |
|  |  | Bonferroni posttests | |  | |  |  |  |  |  |
|  |  | Time point | | Non-epileptic (n = 6) | | Epileptic (n = 7) | Difference | 95% CI | P value |  |
|  |  | pre | | 0.2144 | | 0.2089 | -0.005424 | -0.02633 to 0.01548 | > 0.05 |  |
|  |  | 1d | | 0.2030 | | 0.2116 | 0.008600 | -0.01231 to 0.02951 | > 0.05 |  |
|  |  | 4d | | 0.2131 | | 0.2205 | 0.007338 | -0.01357 to 0.02825 | > 0.05 |  |
|  |  | 8d | | 0.2193 | | 0.2396 | 0.02025 | -0.0006551 to 0.04116 | < 0.05 |  |
|  |  | 16d | | 0.2175 | | 0.2502 | 0.03270 | 0.01179 to 0.05361 | < 0.001 |  |
|  |  | 31d | | 0.2216 | | 0.2480 | 0.02637 | 0.005459 to 0.04727 | < 0.001 |  |
|  |  |  | |  | |  |  |  |  |  |
| **MD (DG)** | **Fig. 5J** | Pearson's correlation. corrected for multiple comparisons | | | | |  |  |  |  |
| **vs.** |  | Time point | Number of X values | | Slope [*10^-9^] | | 95% CI [*10^-9^] | R² value | P value |  |
| **total GCL vol.** |  | pre | 13 | | -0.004 ± 0.003 | | -0.011 to 0.003 | 0.1354 | 1.2972 |  |
|  |  | 1d | 13 | | 0.005 ± 0.006 | | -0.009 to 0.019 | 0.05439 | 2.6592 |  |
|  |  | 4d | 13 | | 0.007 ± 0.005 | | -0.004 to 0.017 | 0.1561 | 1.0884 |  |
|  |  | 8d | 13 | | 0.019 ± 0.007 | | 0.005 to 0.034 | 0.4472 | 0.0744 |  |
|  |  | 16d | 13 | | 0.03 ± 0.007 | | 0.015 to 0.045 | 0.6411 | 0.006 |  |
|  |  | 31d | 13 | | 0.021 ± 0.007 | | 0.006 to 0.035 | 0.4767 | 0.054 |  |
|  |  |  |  | |  | |  |  |  |  |
| **RD (DG)** | **Fig. 5K** | Two-way ANOVA |  | |  | |  |  |  |  |
|  |  | Source of Variation | Interaction | | Time | | Treatment | Subjects |  |  |
|  |  | % of total variation | 8.29 | | 30.54 | | 13.87 | 14.8989 |  |  |
|  |  | P value | 0.0181 | | < 0.0001 | | 0.0084 | 0.0141 |  |  |
|  |  | F value | 3.006 | | 11.08 | | 10.24 | 2.456 |  |  |
|  |  |  |  | |  | |  |  |  |  |
|  |  | Bonferroni posttests |  | |  | |  |  |  |  |
|  |  | Time point | Non-epileptic (n = 6) [*10^-3^] | | Epileptic (n = 7) [*10^-3^] | | Difference [*10^-3^] | 95% CI [*10^-3^] | P value |  |
|  |  | pre | 0.5668 | | 0.5538 | | -0.01291 | -0.07340 to 0.04759 | > 0.05 |  |
|  |  | 1d | 0.5346 | | 0.563 | | 0.02838 | -0.03211 to 0.08887 | > 0.05 |  |
|  |  | 4d | 0.5595 | | 0.5794 | | 0.01989 | -0.04060 to 0.08038 | > 0.05 |  |
|  |  | 8d | 0.5742 | | 0.6216 | | 0.04732 | -0.01317 to 0.1078 | > 0.05 |  |
|  |  | 16d | 0.572 | | 0.6382 | | 0.06619 | 0.005694 to 0.1267 | < 0.01 |  |
|  |  | 31d | 0.5901 | | 0.6355 | | 0.04546 | -0.01503 to 0.1060 | > 0.05 |  |
|  |  |  |  | |  | |  |  |  |  |
| **RD (DG)** | **Fig. 5L** | Pearson's correlation. corrected for multiple comparisons | | | | |  |  |  |  |
| **vs.** |  | Time point | Number of X values | | Slope | | 95% CI | R² value | P value |  |
| **total GCL vol.** |  | pre | 13 | | na | | na | 0.1287 | 1.3722 |  |
|  |  | 1d | 13 | | na | | na | 0.08552 | 1.9932 |  |
|  |  | 4d | 13 | | na | | na | 0.1306 | 1.35 |  |
|  |  | 8d | 13 | | na | | na | 0.2575 | 0.4602 |  |
|  |  | 16d | 13 | | na | | na | 0.3921 | 0.132 |  |
|  |  | 31d | 13 | | na | | na | 0.2244 | 0.612 |  |
|  |  |  |  | |  | |  |  |  |  |
| **AD (DG)** | **Fig. 5M** | Two-way ANOVA |  | |  | |  |  |  |  |
|  |  | Source of Variation | Interaction | | Time | | Treatment | Subjects |  |  |
|  |  | % of total variation | 17.45 | | 37.93 | | 18.50 | 7.1423 |  |  |
|  |  | P value | < 0.0001 | | < 0.0001 | | 0.0002 | 0.0166 |  |  |
|  |  | F value | 12.87 | | 27.96 | | 28.49 | 2.394 |  |  |
|  |  |  |  | |  | |  |  |  |  |
|  |  | Bonferroni posttests |  | |  | |  |  |  |  |
|  |  | Time point | Non-epileptic (n = 6) [*10^-3^] | | Epileptic (n = 7) [*10^-3^] | | Difference [*10^-3^] | 95% CI [*10^-3^] | P value |  |
|  |  | pre | 0.7959 | | 0.7727 | | -0.02322 | -0.1023 to 0.05583 | > 0.05 |  |
|  |  | 1d | 0.7577 | | 0.7783 | | 0.02059 | -0.05846 to 0.09963 | > 0.05 |  |
|  |  | 4d | 0.7992 | | 0.8254 | | 0.02621 | -0.05284 to 0.1053 | > 0.05 |  |
|  |  | 8d | 0.8256 | | 0.9131 | | 0.08753 | 0.008481 to 0.1666 | < 0.01 |  |
|  |  | 16d | 0.8134 | | 0.9756 | | 0.1622 | 0.08313 to 0.2412 | < 0.001 |  |
|  |  | 31d | 0.8143 | | 0.9609 | | 0.1465 | 0.06750 to 0.2256 | < 0.001 |  |
|  |  |  |  | |  | |  |  |  |  |
| **AD (DG)** | **Fig. 5N** | Pearson's correlation. corrected for multiple comparisons | | | | |  |  |  |  |
| **vs.** |  | Time point | Number of X values | | Slope | | 95% CI | R² value | P value |  |
| **total GCL vol.** |  | pre | 13 | | na | | na | 0.1251 | 1.4148 |  |
|  |  | 1d | 13 | | na | | na | 0.01213 | 4.3212 |  |
|  |  | 4d | 13 | | na | | na | 0.1754 | 0.9258 |  |
|  |  | 8d | 13 | | na | | na | 0.6847 | 0.003 |  |
|  |  | 16d | 13 | | na | | na | 0.8419 | < 0.0006 |  |
|  |  | 31d | 13 | | na | | na | 0.6586 | 0.0048 |  |
|  |  |  |  | |  | |  |  |  |  |
| **FA (DG)** | **Fig. 5O** | Two-way ANOVA |  | |  | |  |  |  |  |
|  |  | Source of Variation | Interaction | | Time | | Treatment | Subjects |  |  |
|  |  | % of total variation | 21.40 | | 16.75 | | 6.48 | 15.9358 |  |  |
|  |  | P value | 0.0001 | | 0.0008 | | 0.0581 | 0.0315 |  |  |
|  |  | F value | 6.343 | | 4.965 | | 4.473 | 2.147 |  |  |
|  |  |  |  | |  | |  |  |  |  |
|  |  | Bonferroni posttests |  | |  | |  |  |  |  |
|  |  | Time point | Non-epileptic (n = 6) | | Epileptic (n = 7) | | Difference | 95% CI | P value |  |
|  |  | pre | 0.2180 | | 0.2137 | | -0.004286 | -0.04809 to 0.03952 | > 0.05 |  |
|  |  | 1d | 0.2244 | | 0.2111 | | -0.01329 | -0.05709 to 0.03051 | > 0.05 |  |
|  |  | 4d | 0.2303 | | 0.2274 | | -0.002836 | -0.04664 to 0.04097 | > 0.05 |  |
|  |  | 8d | 0.2385 | | 0.2445 | | 0.006012 | -0.03779 to 0.04981 | > 0.05 |  |
|  |  | 16d | 0.2257 | | 0.2707 | | 0.04494 | 0.001139 to 0.08874 | < 0.05 |  |
|  |  | 31d | 0.2077 | | 0.2659 | | 0.05826 | 0.01446 to 0.1021 | < 0.001 |  |
|  |  |  |  | |  | |  |  |  |  |
| **FA (DG)** | **Fig. 5P** | Pearson's correlation. corrected for multiple comparisons | | | | |  |  |  |  |
| **vs.** |  | Time point | Number of X values | | Slope [*10^-9^] | | 95% CI [*10-9] | R² value | P value |  |
| **total GCL vol.** |  | pre | 13 | | -0.002 ± 0.007 | | -0.016 to 0.013 | 0.005804 | 4.8276 |  |
|  |  | 1d | 13 | | -0.012 ± 0.008 | | -0.029 to 0.005 | 0.1881 | 0.8322 |  |
|  |  | 4d | 13 | | na ± 0.009 | | -0.02 to 0.019 | 0.0002320 | 5.7636 |  |
|  |  | 8d | 13 | | 0.016 ± 0.016 | | -0.02 to 0.053 | 0.08246 | 2.0484 |  |
|  |  | 16d | 13 | | 0.057 ± 0.009 | | 0.037 to 0.077 | 0.7804 | < 0.0006 |  |
|  |  | 31d | 13 | | 0.055 ± 0.012 | | 0.029 to 0.08 | 0.6687 | 0.0036 |  |
|  |  |  |  | |  | |  |  |  |  |
| **MD (DG)** | **Fig. 5I** | Two-way ANOVA |  | |  | |  |  |  |  |
|  |  | Source of Variation | Interaction | | Time | | Treatment | Subjects |  |  |
|  |  | % of total variation | 11.31 | | 44.66 | | 0.26 | 13.8792 |  |  |
|  |  | P value | 0.0013 | | < 0.0001 | | 0.6594 | 0.0095 |  |  |
|  |  | F value | 4.677 | | 18.47 | | 0.2052 | 2.609 |  |  |
|  |  |  |  | |  | |  |  |  |  |
|  |  | Bonferroni posttests |  | |  | |  |  |  |  |
|  |  | Time point | Non-epileptic (n = 6) [*10^-3^] | | Epileptic (n = 7) [*10^-3^] | | Difference [*10^-3^] | 95% CI [*10^-3^] | P value |  |
|  |  | pre | 0.6935 | | 0.6666 | | -0.02690 | -0.09551 to 0.04171 | > 0.05 |  |
|  |  | 1d | 0.6469 | | 0.6298 | | -0.01706 | -0.08567 to 0.05155 | > 0.05 |  |
|  |  | 4d | 0.6759 | | 0.6504 | | -0.02548 | -0.09409 to 0.04313 | > 0.05 |  |
|  |  | 8d | 0.7077 | | 0.7015 | | -0.006122 | -0.07473 to 0.06249 | > 0.05 |  |
|  |  | 16d | 0.698 | | 0.7456 | | 0.04762 | -0.02099 to 0.1162 | > 0.05 |  |
|  |  | 31d | 0.7109 | | 0.7707 | | 0.05977 | -0.008843 to 0.1284 | > 0.05 |  |
|  |  |  |  | |  | |  |  |  |  |
| **MD (CA1)** | **Fig. 5-S1A** | Pearson's correlation. corrected for multiple comparisons | | | | |  |  |  |  |
| **vs.** |  | Time point | Number of X values | | Slope [*10^-5^] | | 95% CI [*10^-5^] | R² value | P value |  |
| **microgliosis** |  | pre | 13 | | -0.3984 ± 0.2081 | | -0.8565 to 0.0596 | 0.2499 | 0.4914 |  |
|  |  | 1d | 13 | | -0.3995 ± 0.2719 | | -0.9979 to 0.1989 | 0.1641 | 1.0188 |  |
|  |  | 4d | 13 | | -0.4113 ± 0.3279 | | -1.133 to 0.3105 | 0.1251 | 1.4148 |  |
|  |  | 8d | 13 | | -0.1667 ± 0.3013 | | -0.8299 to 0.4965 | 0.02707 | 3.5472 |  |
|  |  | 16d | 13 | | 0.6909 ± 0.2771 | | 0.081 to 1.301 | 0.3611 | 0.1794 |  |
|  |  | 31d | 13 | | 0.7167 ± 0.2904 | | 0.0775 to 1.356 | 0.3563 | 0.1872 |  |
|  |  |  |  | |  | |  |  |  |  |
| **AD (CA1)** | **Fig. 5-S1B** | Two-way ANOVA |  | |  | |  |  |  |  |
|  |  | Source of Variation | Interaction | | Time | | Treatment | Subjects |  |  |
|  |  | % of total variation | 16.52 | | 47.71 | | 0.01 | 10.7221 |  |  |
|  |  | P value | < 0.0001 | | < 0.0001 | | 0.9266 | 0.0105 |  |  |
|  |  | F value | 8.713 | | 25.16 | | 0.008896 | 2.570 |  |  |
|  |  |  |  | |  | |  |  |  |  |
|  |  | Bonferroni posttests |  | |  | |  |  |  |  |
|  |  | Time point | Non-epileptic (n = 6) [*10^-3^] | | Epileptic (n = 7) [*10^-3^] | | Difference [*10^-3^] | 95% CI [*10^-3^] | P value |  |
|  |  | pre | 0.8301 | | 0.8031 | | -0.02693 | -0.1015 to 0.04763 | > 0.05 |  |
|  |  | 1d | 0.7809 | | 0.7362 | | -0.04464 | -0.1192 to 0.02992 | > 0.05 |  |
|  |  | 4d | 0.8188 | | 0.776 | | -0.04288 | -0.1174 to 0.03168 | > 0.05 |  |
|  |  | 8d | 0.8589 | | 0.8339 | | -0.02504 | -0.09960 to 0.04952 | > 0.05 |  |
|  |  | 16d | 0.8356 | | 0.8952 | | 0.05959 | -0.01497 to 0.1341 | > 0.05 |  |
|  |  | 31d | 0.849 | | 0.936 | | 0.08706 | 0.01250 to 0.1616 | < 0.01 |  |
|  |  |  |  | |  | |  |  |  |  |
| **AD (CA1)** | **Fig. 5-S1C** | Pearson's correlation. corrected for multiple comparisons | | | | |  |  |  |  |
| **vs.** |  | Time point | Number of X values | | Slope [*10^-5^] | | 95% CI [*10^-5^] | R² value | P value |  |
| **microgliosis** |  | pre | 13 | | -0.3962 ± 0.2633 | | -0.9758 to 0.1833 | 0.1707 | 0.963 |  |
|  |  | 1d | 13 | | -0.7593 ± 0.2284 | | -1.262 to -0.2565 | 0.5011 | 0.0408 |  |
|  |  | 4d | 13 | | -0.7135 ± 0.3326 | | -1.445 to 0.0185 | 0.2950 | 0.3306 |  |
|  |  | 8d | 13 | | -0.4109 ± 0.296 | | -1.062 to 0.2405 | 0.1491 | 1.155 |  |
|  |  | 16d | 13 | | 0.8787 ± 0.2946 | | 0.2304 to 1.527 | 0.4472 | 0.075 |  |
|  |  | 31d | 13 | | 1.115 ± 0.3751 | | 0.2893 to 1.941 | 0.4454 | 0.0762 |  |
|  |  |  |  | |  | |  |  |  |  |
| **RD (CA1)** | **Fig. 5-S1D** | Two-way ANOVA |  | |  | |  |  |  |  |
|  |  | Source of Variation | Interaction | | Time | | Treatment | Subjects |  |  |
|  |  | % of total variation | 8.39 | | 40.16 | | 0.60 | 16.6487 |  |  |
|  |  | P value | 0.0208 | | < 0.0001 | | 0.5426 | 0.0089 |  |  |
|  |  | F value | 2.920 | | 13.97 | | 0.3949 | 2.633 |  |  |
|  |  |  |  | |  | |  |  |  |  |
|  |  | Bonferroni posttests |  | |  | |  |  |  |  |
|  |  | Time point | Non-epileptic (n = 6) [*10^-3^] | | Epileptic (n = 7) [*10^-3^] | | Difference [*10^-3^] | 95% CI [*10^-3^] | P value |  |
|  |  | pre | 0.6252 | | 0.5983 | | -0.02689 | -0.09530 to 0.04152 | > 0.05 |  |
|  |  | 1d | 0.5799 | | 0.5766 | | -0.003266 | -0.07168 to 0.06514 | > 0.05 |  |
|  |  | 4d | 0.6044 | | 0.5877 | | -0.01679 | -0.08520 to 0.05162 | > 0.05 |  |
|  |  | 8d | 0.632 | | 0.6354 | | 0.003335 | -0.06507 to 0.07174 | > 0.05 |  |
|  |  | 16d | 0.6292 | | 0.6708 | | 0.04164 | -0.02677 to 0.1100 | > 0.05 |  |
|  |  | 31d | 0.6419 | | 0.688 | | 0.04612 | -0.02229 to 0.1145 | > 0.05 |  |
|  |  |  |  | |  | |  |  |  |  |
| **RD (CA1)** | **Fig. 5-S1E** | Pearson's correlation. corrected for multiple comparisons | | | | |  |  |  |  |
| **vs.** |  | Time point | Number of X values | | Slope [*10^-5^] | | 95% CI [*10^-5^] | R² value | P value |  |
| **microgliosis** |  | pre | 13 | | -0.3996 ± 0.1826 | | -0.8014 to 0.0023 | 0.3033 | 0.3066 |  |
|  |  | 1d | 13 | | -0.2196 ± 0.2976 | | -0.8747 to 0.4355 | 0.04716 | 2.8566 |  |
|  |  | 4d | 13 | | -0.2602 ± 0.3342 | | -0.9957 to 0.4754 | 0.05221 | 2.7162 |  |
|  |  | 8d | 13 | | -0.0446 ± 0.3224 | | -0.7541 to 0.665 | 0.001735 | 5.355 |  |
|  |  | 16d | 13 | | 0.597 ± 0.275 | | -0.0082 to 1.202 | 0.3000 | 0.3162 |  |
|  |  | 31d | 13 | | 0.5176 ± 0.2603 | | -0.0552 to 1.091 | 0.2645 | 0.4332 |  |
|  |  |  |  | |  | |  |  |  |  |
| **FA (CA1)** | **Fig. 5-S1F** | Two-way ANOVA |  | |  | |  |  |  |  |
|  |  | Source of Variation | Interaction | | Time | | Treatment | Subjects |  |  |
|  |  | % of total variation | 24.05 | | 6.57 | | 3.17 | 22.3001 |  |  |
|  |  | P value | 0.0001 | | 0.1495 | | 0.2372 | 0.0091 |  |  |
|  |  | F value | 6.229 | | 1.702 | | 1.563 | 2.626 |  |  |
|  |  |  |  | |  | |  |  |  |  |
|  |  | Bonferroni posttests |  | |  | |  |  |  |  |
|  |  | Time point | Non-epileptic (n = 6) | | Epileptic (n = 7) | | Difference | 95% CI | P value |  |
|  |  | pre | 0.1878 | | 0.1928 | | 0.004981 | -0.03142 to 0.04138 | > 0.05 |  |
|  |  | 1d | 0.1977 | | 0.1624 | | -0.03522 | -0.07162 to 0.001173 | < 0.05 |  |
|  |  | 4d | 0.2038 | | 0.1850 | | -0.01878 | -0.05518 to 0.01761 | > 0.05 |  |
|  |  | 8d | 0.2069 | | 0.1786 | | -0.02824 | -0.06463 to 0.008161 | > 0.05 |  |
|  |  | 16d | 0.1892 | | 0.1935 | | 0.004376 | -0.03202 to 0.04077 | > 0.05 |  |
|  |  | 31d | 0.1852 | | 0.2114 | | 0.02620 | -0.01020 to 0.06259 | > 0.05 |  |
|  |  |  |  | |  | |  |  |  |  |
| **FA (CA1)**  **vs.** | **Fig. 5-S1G** | Pearson's correlation. corrected for multiple comparisons | | | | |  |  |  |  |
| **microgliosis** |  | Time point | Number of X values | | Slope [*10^-6^] | | 95% CI [*10^-6^] | R² value | P value |  |
|  |  | pre | 13 | | 0.08434 ± 0.5442 | | -0.3545 to 2.041 | 0.1792 | 0.897 |  |
|  |  | 1d | 13 | | -3.987 ± 1.727 | | -7.788 to -0.1859 | 0.3264 | 0.2484 |  |
|  |  | 4d | 13 | | -3.246 ± 1.736 | | -7.066 to 0.5736 | 0.2413 | 0.5292 |  |
|  |  | 8d | 13 | | -3.446 ± 2.161 | | -8.203 to 1.310 | 0.1878 | 0.8346 |  |
|  |  | 16d | 13 | | 0.9670 ± 1.211 | | -1.698 to 3.632 | 0.05481 | 2.6484 |  |
|  |  | 31d | 13 | | 4.059 ± 1.232 | | 1.346 to 6.771 | 0.4965 | 0.0432 |  |
|  |  |  |  | |  | |  |  |  |  |
| **dvD (DG)** | **Fig. 6F** | Two-way ANOVA |  | |  | |  |  |  |  |
|  |  | Source of Variation | Interaction | | Time | | Treatment | Subjects |  |  |
|  |  | % of total variation | 15.26 | | 27.44 | | 23.28 | 12.5197 |  |  |
|  |  | P value | < 0.0001 | | < 0.0001 | | 0.0009 | 0.0012 |  |  |
|  |  | F value | 9.120 | | 16.40 | | 20.45 | 3.401 |  |  |
|  |  |  |  | |  | |  |  |  |  |
|  |  | Bonferroni posttests |  | |  | |  |  |  |  |
|  |  | Time point | Non-epileptic (n = 6) [*10^-3^] | | Epileptic (n = 7) [*10^-3^] | | Difference [*10^-3^] | 95% CI [*10^-3^] | P value |  |
|  |  | pre | 0.6789 | | 0.6443 | | -0.03461 | -0.1436 to 0.07438 | > 0.05 |  |
|  |  | 1d | 0.6249 | | 0.6694 | | 0.04448 | -0.06450 to 0.1535 | > 0.05 |  |
|  |  | 4d | 0.6434 | | 0.7078 | | 0.06434 | -0.04464 to 0.1733 | > 0.05 |  |
|  |  | 8d | 0.6639 | | 0.8079 | | 0.144 | 0.03504 to 0.2530 | < 0.001 |  |
|  |  | 16d | 0.6754 | | 0.855 | | 0.1795 | 0.07054 to 0.2885 | < 0.001 |  |
|  |  | 31d | 0.6945 | | 0.8452 | | 0.1507 | 0.04167 to 0.2596 | < 0.001 |  |
|  |  |  |  | |  | |  |  |  |  |
| **GFAP dens.** | **Fig. 6I** | One-way ANOVA |  | |  | |  |  |  |  |
|  |  | P value | < 0.0001 | |  | |  |  |  |  |
|  |  | Number of groups | 9 | |  | |  |  |  |  |
|  |  | F value | 45.83 | |  | |  |  |  |  |
|  |  | R² value | 0.7942 | |  | |  |  |  |  |
|  |  |  |  | |  | |  |  |  |  |
|  |  | Bonferroni's Multiple Comparison Test | | |  | |  |  |  |  |
|  |  | Groups (n*) | | Mean Diff. | 95% CI | | P values |  |  |  |
|  |  | controls (40) vs NP27 (8) | | -3.275 | -5.694 to -0.8559 | | < 0.001 |  |  |  |
|  |  | controls (40) vs NP10 (8) | | -2.177 | -4.596 to 0.2426 | | > 0.05 |  |  |  |
|  |  | controls (40) vs NP11 (8) | | -6.063 | -8.482 to -3.644 | | < 0.001 |  |  |  |
|  |  | controls (40) vs NP26 (8) | | -7.358 | -9.777 to -4.939 | | < 0.001 |  |  |  |
|  |  | controls (40) vs NP31 (8) | | -8.143 | -10.56 to -5.724 | | < 0.001 |  |  |  |
|  |  | controls (40) vs NP14 (8) | | -7.234 | -9.653 to -4.815 | | < 0.001 |  |  |  |
|  |  | controls (40) vs NP34 (8) | | -8.582 | -11.00 to -6.163 | | < 0.001 |  |  |  |
|  |  | controls (40) vs NP25 (8) | | -8.107 | -10.53 to -5.688 | | < 0.001 |  |  |  |
|  |  | NP27 (8) vs NP10 (8) | | 1.098 | -2.025 to 4.222 | | > 0.05 |  |  |  |
|  |  | NP27 (8) vs NP11 (8) | | -2.788 | -5.911 to 0.3354 | | > 0.05 |  |  |  |
|  |  | NP27 (8) vs NP26 (8) | | -4.083 | -7.206 to -0.9599 | | < 0.01 |  |  |  |
|  |  | NP27 (8) vs NP31 (8) | | -4.868 | -7.991 to -1.745 | | < 0.001 |  |  |  |
|  |  | NP27 (8) vs NP14 (8) | | -3.959 | -7.082 to -0.8362 | | < 0.01 |  |  |  |
|  |  | NP27 (8) vs NP34 (8) | | -5.307 | -8.430 to -2.184 | | < 0.001 |  |  |  |
|  |  | NP27 (8) vs NP25 (8) | | -4.832 | -7.955 to -1.709 | | < 0.001 |  |  |  |
|  |  | NP10 (8) vs NP11 (8) | | -3.886 | -7.009 to -0.7631 | | < 0.01 |  |  |  |
|  |  | NP10 (8) vs NP26 (8) | | -5.181 | -8.305 to -2.058 | | < 0.001 |  |  |  |
|  |  | NP10 (8) vs NP31 (8) | | -5.966 | -9.089 to -2.843 | | < 0.001 |  |  |  |
|  |  | NP10 (8) vs NP14 (8) | | -5.058 | -8.181 to -1.935 | | < 0.001 |  |  |  |
|  |  | NP10 (8) vs NP34 (8) | | -6.406 | -9.529 to -3.283 | | < 0.001 |  |  |  |
|  |  | NP10 (8) vs NP25 (8) | | -5.931 | -9.054 to -2.808 | | < 0.001 |  |  |  |
|  |  | NP11 (8) vs NP26 (8) | | -1.295 | -4.418 to 1.828 | | > 0.05 |  |  |  |
|  |  | NP11 (8) vs NP31 (8) | | -2.080 | -5.203 to 1.043 | | > 0.05 |  |  |  |
|  |  | NP11 (8) vs NP14 (8) | | -1.172 | -4.295 to 1.951 | | > 0.05 |  |  |  |
|  |  | NP11 (8) vs NP34 (8) | | -2.520 | -5.643 to 0.6034 | | > 0.05 |  |  |  |
|  |  | NP11 (8) vs NP25 (8) | | -2.045 | -5.168 to 1.078 | | > 0.05 |  |  |  |
|  |  | NP26 (8) vs NP31 (8) | | -0.7846 | -3.908 to 2.338 | | > 0.05 |  |  |  |
|  |  | NP26 (8) vs NP14 (8) | | 0.1237 | -2.999 to 3.247 | | > 0.05 |  |  |  |
|  |  | NP26 (8) vs NP34 (8) | | -1.224 | -4.347 to 1.899 | | > 0.05 |  |  |  |
|  |  | NP26 (8) vs NP25 (8) | | -0.7493 | -3.872 to 2.374 | | > 0.05 |  |  |  |
|  |  | NP31 (8) vs NP14 (8) | | 0.9084 | -2.215 to 4.031 | | > 0.05 |  |  |  |
|  |  | NP31 (8) vs NP34 (8) | | -0.4396 | -3.563 to 2.683 | | > 0.05 |  |  |  |
|  |  | NP31 (8) vs NP25 (8) | | 0.03537 | -3.088 to 3.158 | | > 0.05 |  |  |  |
|  |  | NP14 (8) vs NP34 (8) | | -1.348 | -4.471 to 1.775 | | > 0.05 |  |  |  |
|  |  | NP14 (8) vs NP25 (8) | | -0.8730 | -3.996 to 2.250 | | > 0.05 |  |  |  |
|  |  | NP34 (8) vs NP25 (8) | | 0.4750 | -2.648 to 3.598 | | > 0.05 |  |  |  |
|  |  |  | |  |  | |  |  |  |  |
| **dvD (DG)** | **Fig 6G** | Pearson's correlation. corrected for multiple comparisons | | | | |  |  |  |  |
| **vs.** |  | Time point | | Number of X values | | Slope | 95% CI | R² value | P value |  |
| **total GCL vol.** |  | pre | | 13 | | na | na | 0.1367 | 1.2822 |  |
|  |  | 1d | | 13 | | na | na | 0.03742 | 3.1596 |  |
|  |  | 4d | | 13 | | na | na | 0.5621 | 0.0192 |  |
|  |  | 8d | | 13 | | na | na | 0.6391 | 0.006 |  |
|  |  | 16d | | 13 | | na | na | 0.7889 | < 0.0006 |  |
|  |  | 31d | | 13 | | na | na | 0.6399 | 0.006 |  |
|  |  |  | |  | |  |  |  |  |  |
| **dvD vol. (DG)** | **Fig. 6J** | Pearson's correlation. corrected for multiple comparisons | | | | |  |  |  |  |
| **vs.** |  | Time point | | Number of X values | | Slope | 95% CI | R² value | P value |  |
| **integr. GFAP dens.** |  | pre | | 13 | | -0.02457 ± 0.02280 | -0.07476 to 0.02562 | 0.09547 | 1.8258 |  |
|  |  | 1d | | 13 | | 0.004600 ± 0.01658 | -0.03190 to 0.04110 | 0.006944 | 4.7202 |  |
|  |  | 4d | | 13 | | 0.07367 ± 0.01782 | 0.03446 to 0.1129 | 0.6085 | 0.0102 |  |
|  |  | 8d | | 13 | | 0.2218 ± 0.02849 | 0.1591 to 0.2845 | 0.8464 | < 0.0006 |  |
|  |  | 16d | | 13 | | 0.3651 ± 0.04230 | 0.2720 to 0.4582 | 0.8713 | < 0.0006 |  |
|  |  | 31d | | 13 | | 0.3529 ± 0.05146 | 0.2396 to 0.4662 | 0.8104 | < 0.0006 |  |
|  |  |  | |  | |  |  |  |  |  |
| **FA (DG)** | **Fig. 7P** | Pearson's correlation | |  | |  |  |  |  |  |
| **vs.** |  | Time point | | Number of X values | | Slope [*10^-3^] | 95% CI [*10^-3^] | R² value | P value |  |
| **GFAP dens.** |  | na | | 5 | | 12.88 ± 3.926 | 0.3834 to 25.37 | 0.7819 | 0.0464 |  |
|  |  |  | |  | |  |  |  |  |  |
| **FA (DG)** | **Fig. 7Q** | Pearson's correlation | |  | |  |  |  |  |  |
| **vs.** |  | Time point | | Number of X values | | Slope [*10^-3^] | 95% CI [*10^-3^] | R² value | P value |  |
| **Synpo dens.** |  | na | | 5 | | 3.746 ± 1.809 | -2.012 to 9.503 | 0.5882 | 0.1302 |  |
|  |  |  | |  | |  |  |  |  |  |
| **PC1 score** | **Fig. 8B** | Pearson's correlation | |  | |  |  |  |  |  |
| **vs.** |  | Time point | | Number of X values | | Slope [*10^-9^] | 95% CI [*10^-9^] | R² value | P value |  |
| **total GCL vol.** |  | 36d | | 7 | | 0.119 ± 0.021 | 0.064 to 0.174 | 0.8611 | 0.0026 |  |
|  |  |  | |  | |  |  |  |  |  |
| **PC1 score** | **Fig. 8B** | Pearson's correlation | |  | |  |  |  |  |  |
| **vs.** |  | Time point | | Number of X values | | Slope [*10^-3^] | 95% CI [*10^-3^] | R² value | P value |  |
| **microgliosis** |  | 36d | | 7 | | 0.3506 ± 0.1247 | 0.03008 to 0.6711 | 0.6127 | 0.0374 |  |
|  |  |  | |  | |  |  |  |  |  |
| **PC1 score** | **Fig. 8B** | Pearson's correlation | |  | |  |  |  |  |  |
| **vs.** |  | Time point | | Number of X values | | Slope | 95% CI | R² value | P value |  |
| **radial gliosis** |  | 36d | | 7 | | 0.3784 ± 0.1771 | -0.07705 to 0.8338 | 0.4771 | 0.0858 |  |
|  |  |  | |  | |  |  |  |  |  |
| **seiz-like freq.** | **Fig. 1C** | One-way ANOVA | |  | |  |  |  |  |  |
|  |  | P value | | 0.0028 | |  |  |  |  |  |
|  |  | Number of groups | | 6 | |  |  |  |  |  |
|  |  | F value | | 4.826 | |  |  |  |  |  |
|  |  | R² values | | 0.4720 | |  |  |  |  |  |
|  |  |  | |  | |  |  |  |  |  |
|  |  | Bonferroni's Multiple Comparison Test | | | |  |  |  |  |  |
|  |  | Groups (n°) | | Mean Diff. | | 95% CI | P values |  |  |  |
|  |  | NP27 (5) vs NP11 (7) | | 0.004694 | | -0.3690 to 0.3784 | > 0.05 |  |  |  |
|  |  | NP27 (5) vs NP31 (5) | | 0.09587 | | -0.3078 to 0.4995 | > 0.05 |  |  |  |
|  |  | NP27 (5) vs NP14 (4) | | 0.2605 | | -0.1676 to 0.6887 | > 0.05 |  |  |  |
|  |  | NP27 (5) vs NP34 (5) | | 0.2822 | | -0.1215 to 0.6858 | > 0.05 |  |  |  |
|  |  | NP27 (5) vs NP25 (7) | | 0.4358 | | 0.06208 to 0.8095 | < 0.05 |  |  |  |
|  |  | NP11 (7) vs NP31 (5) | | 0.09118 | | -0.2825 to 0.4649 | > 0.05 |  |  |  |
|  |  | NP11 (7) vs NP14 (4) | | 0.2559 | | -0.1442 to 0.6559 | > 0.05 |  |  |  |
|  |  | NP11 (7) vs NP34 (5) | | 0.2775 | | -0.09626 to 0.6512 | > 0.05 |  |  |  |
|  |  | NP11 (7) vs NP25 (7) | | 0.4311 | | 0.08994 to 0.7723 | < 0.01 |  |  |  |
|  |  | NP31 (5) vs NP14 (4) | | 0.1647 | | -0.2635 to 0.5928 | > 0.05 |  |  |  |
|  |  | NP31 (5) vs NP34 (5) | | 0.1863 | | -0.2174 to 0.5900 | > 0.05 |  |  |  |
|  |  | NP31 (5) vs NP25 (7) | | 0.3399 | | -0.03379 to 0.7137 | > 0.05 |  |  |  |
|  |  | NP14 4) vs NP34 (5) | | 0.02161 | | -0.4065 to 0.4498 | > 0.05 |  |  |  |
|  |  | NP14 (4) vs NP25 (7) | | 0.1753 | | -0.2248 to 0.5753 | > 0.05 |  |  |  |
|  |  | NP34 (5) vs NP25 (7) | | 0.1536 | | -0.2201 to 0.5274 | > 0.05 |  |  |  |
|  |  |  | |  | |  |  |  |  |  |
| **PC1 score** | **Fig. 9C** | Pearson's correlation | |  | |  |  |  |  |  |
| **vs.** |  | Time point | | Number of X values | | Slope | 95% CI | R² value | P value |  |
| **seiz.-like freq.** |  | 36d | | 6 | | -6.005 ± 0.7853 | -8.185 to -3.825 | 0.9360 | 0.0016 |  |
|  |  |  | |  | |  |  |  |  |  |

**Supplementary File 1: Quantitative summary of statistically tested parameters.** The table displays all results statistical tests performed (right column) for each parameter (left column). The reference to the corresponding figure is given in the middle column. CI, confidence interval; n, number of animals; n*, number of sections; n°, number of recordings.
